# Prospects for Malaria Vaccines: Pre-Erythrocytic Stages, Blood Stages, and Transmission-Blocking Stages

**DOI:** 10.1155/2019/9751471

**Published:** 2019-10-03

**Authors:** Jingtong Zheng, He Pan, Yinuo Gu, Xu Zuo, Nan Ran, Yuze Yuan, Chao Zhang, Fang Wang

**Affiliations:** Department of Pathogenobiology, College of Basic Medicine, Jilin University, Changchun 130000, China

## Abstract

Malaria is a disease of public health importance in many parts of the world. Currently, there is no effective way to eradicate malaria, so developing safe, efficient, and cost-effective vaccines against this disease remains an important goal. Current research on malaria vaccines is focused on developing vaccines against pre-erythrocytic stage parasites and blood-stage parasites or on developing a transmission-blocking vaccine. Here, we briefly describe the progress made towards a vaccine against *Plasmodium falciparum*, the most pathogenic of the malaria parasite species to infect humans.

## 1. Introduction

**Figure 1:**
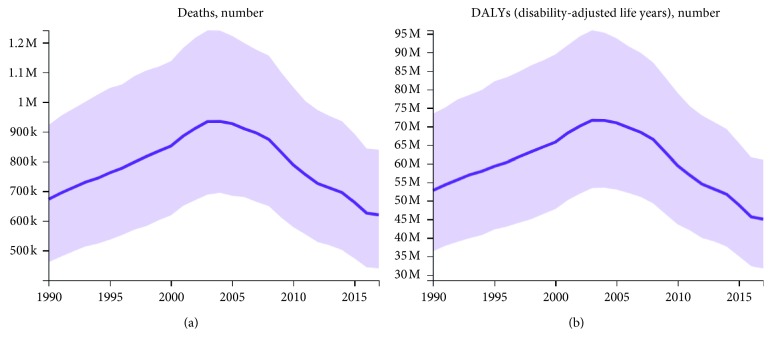
Deaths of malaria (a) and DALYs of malaria (b) between 1990 and 2017, all ages (created with data from the Global Burden of Disease Study 2017 (GBD 2017) results).

Malaria caused by *Plasmodium falciparum* (*Pf*) is a parasitic disease whose pathology seriously endangers human health. In 2017, 219 million people were infected with this parasite species and about 435,000 people, mostly children under the age of 5 years, died from infections with it (Figure 1).

In 2015, the World Health Organization (WHO) endorsed a new Global Technical Strategy for Malaria (http://www.who.int/). This strategy includes ambitious goals for malaria control and elimination over the next 15 years. A key target is the elimination of malaria in at least 10 countries by 2020. The WHO has recently reported on the progress made and challenges outstanding in 21 countries (called the E-2020 countries), which identified it in 2016 as having the potential to become malaria free by 2020 (http://www.who.int/). Although remarkable achievements in malaria control have been made in recent years, serious difficulties persist because of the emergence and spread of parasite resistance to antimalarial drugs [1]. Moreover, development of resistance to pyrethroid insecticides has enabled the mosquito vectors of malaria to survive when they make contact with long-lasting insecticide-treated mosquito nets [2]. The development of a safe, inexpensive, and effective vaccine is widely believed to be critical for the control and eradication of malaria. The WHO, United Nations Development Program, and the World Bank have identified malaria vaccine research as one of the top three global priorities for vaccine development. Much research effort has been directed at the following four different approaches towards a malaria vaccine: a vaccine targeting the whole organism, a live attenuated vaccine, a genetically engineered vaccine, and a subunit vaccine, but no widely accepted vaccine has been developed to date. Recently, some studies investigating the effects of *Pf* sporozoite (SPZ) vaccines have reported that they hold great promise for protection in humans, but larger sample sizes are needed to validate their protective effects [3, 4]. Researchers face challenges increasing vaccine antigenicity while addressing safety issue as well.

According to the life history of *Pf* in humans, malaria vaccines can be divided into three types (Table 1): pre-erythrocytic, blood-stage, and transmission-blocking vaccines (TBVs). Vaccine candidates are being tested in clinical trials where several have shown potential. Here, we briefly introduce the progress made in the development of a vaccine against *Pf* and the current advances being made in this field.

## 2. Pre-Erythrocytic Vaccines

**Figure 2:**
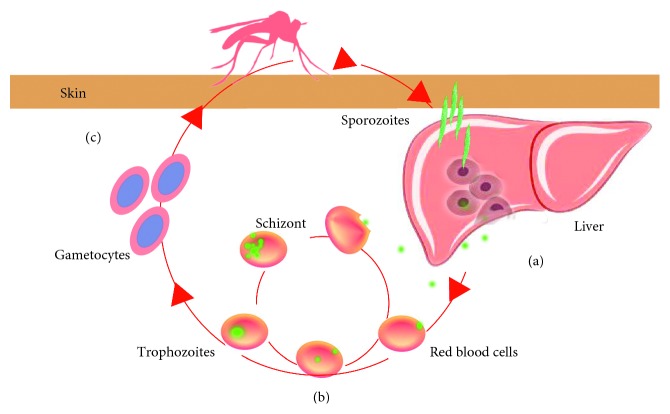
Vaccines target the life cycle of *Plasmodium falciparum*. (a) Pre-erythrocytic *Plasmodium* vaccine: vaccine produces antibodies that kill infected liver cells or interfere with the malaria parasite during liver cell proliferation; (b) asexual blood-stage vaccine: vaccine aims primarily at reducing parasite load or eliminating circulating parasites; (c) vaccine aims at controlling the transmission of malaria parasites from human hosts to the mosquito vectors.

Pre-erythrocytic vaccines, also known as anti-infective vaccines, mainly target the spores of *Pf*. They induce specific antibodies that kill infected liver cells or interfere with the malaria parasite during liver cell proliferation, thereby blocking the release of infectious merozoites and achieving an anti-infection effect (Figure 2(a)). Vaccines targeting pre-erythrocytic *Plasmodium* must have a 100% protection rate to achieve a real protective effect. At present, most pre-erythrocytic malaria vaccine research is focused on the development of subunit vaccines against parasite proteins such as the *Pf* circumsporozoite protein (PfCSP), the thrombospondin-related adhesion protein (TRAP), and the liver stage antigen (LSA).

### 2.1. PfCSP Vaccines

Located on the surface of the mature sporozoite as a 40–60 kDa pre-erythroid antigen, PfCSP plays a key role in sporozoite invasion of liver cells [5]. Till now, DNA vaccine against CSP has been studied for years, which is considered to be a simple and stable vaccine, but the situation of the DNA vaccine in the human body is still in the stage of development and testing. Therefore, in order to better induce an effective immune response, the DNA vaccine gp96NTD-CSP was designed. Heat-shock protein (HSP) is designed to induce dendritic cell maturation and promote cross-antigen presentation, making it is an important immune adjuvant and immune delivery system. gp96 of HSP can be effectively presented to major histocompatibility complex I, leading to CD8^+^ T-cell activation. Tan et al. [6] reported that the adjuvant properties of gp96NTD enhanced the immunogenicity and protective efficacy of this vaccine by inducing high levels of CSP-specific antibodies and a strong CD8^+^ T-cell response. It is reported that CSP-based structures induce high levels of protection in mice, but they are less immunogenic in humans. The reason may be the lack of antibodies caused by DNA vaccine itself or the inability of T-cell immune response to completely eliminate parasites.

#### 2.1.1. RTS,S/AS01 and RTS,S/AS02

RTS,S/AS01 is a current lead recombinant candidate vaccine against malaria [30]. RTS,S is the vaccine with epitopes R and T of B and T cells recombined to the C-terminal end of CSP of PF 3D7, membrane surface protein(S) of hepatitis virus C, and free copy of protein S. AS01 is the adjuvant system to increase the immune system response. Protective immune responses after vaccination with RTS,S are dependent primarily on antibody responses against the central repeat region [31–33]. The RTS,S/AS01 vaccine, which was found to protect African children against clinical and severe malaria [7], has been tested in a phase III trial [8] and has received a positive rating from the European Medicines Agency, but it may not be effective against *Pf* isolates from southern and central Africa transmission-blocking vaccines or other global regions. Since the C-terminal region contains some important T-cell epitopes [9] which related to CD4^+^ T-cell responses, the high genetic diversity in the C-terminal region of the PfCSP molecule may lead to the lack of overall protective efficacy. Therefore, genetic diversity assessment of the C-terminal region of PfCSP is an important aspect of developing an RTS,S/AS01 vaccine for widespread use. Moreover, a phase III trial indicates that the C-terminal sequence-unmatched strains show lower efficacy [10]. To evaluate the clinical malaria incidences and serious adverse events, 3084 children (aged 3–7 years) were enrolled in the phase III trial for 3-year studies. In the studies, primary outcome is the occurrence of severe malaria meeting the primary case definition, and secondary outcome includes the occurrence of clinical malaria meeting the primary and secondary case definitions, occurrence of malaria hospitalization meeting each of the case definitions, prevalence of anemia, and prevalence of parasitemia. It is reported that 66 of the severe malaria cases were reported in 3-year studies. Moreover, clinical malaria incidences in the four-dose, three-dose, and control groups were 1.079 PPY, 1.108 PPY, and 1.016 PPY, respectively [11].

Another candidate vaccine was that RTS,S combined with adjuvant systems AS02 named RTS,S/AS02 [30]. It is reported that both RTS,S/AS01 and RTS,S/AS02 vaccines exhibited better CS-specific immune responses than nonadjuvanted RTS,S. But the anti-CS antibody response to RTS,S/AS01 was much better than RTS,S/AS02 in studies in animals and phase IIa CHMI trial conducted at WRAIR, which might be one of the reasons that RTS,S/AS01 was much widely used than RTS,S/AS02.

#### 2.1.2. PfCSP Bacteria Vaccines

In addition, vectors like viruses and bacteria can be also used as malaria vaccines. Because these vectors are based on the whole organism, they usually do not need assistance to stimulate the innate immune system compared with DNA vaccines. Bacterial vectors are one of the most common delivery vectors for malaria vaccines. *Salmonella enterica* serovar Typhi live vector vaccines have been used widely since 2009 [12]; in 1997, Gonzalez et al. [13] inserted the PfCSP gene into the pUC19 vector to create pUC-PfCSP. The construct was recombined with pKK233-3 to obtain pKK233-PfCSP and then transformed into the *S. enterica* serovar Typhi strain CVD 908 by electroporation. The resultant CVD 908 omega strain (delta aroC1019::tacP-rcsp) was well tolerated by 10 volunteers who each received two doses of 5 × 10^7^ organisms 8 days apart. The volunteers showed a fourfold rise in their antibody levels against a recombinant portion of CSP, and CSP-specific CD8^+^ cytotoxic T lymphocyte activity was induced [13].

### 2.2. TRAP Vaccines

TRAP, also known as the *Pf*SPZ surface protein 2 (SSP-2), is a major antigen that plays an important role in SPZ invasion of mosquito salivary glands and hepatocytes [14]. Recently, researchers have found that TRAP interacts directly with integrins on the surfaces of human cells [34]. The multiepitope (ME) string TRAP antigen contains a fusion protein of ME, followed by pre-erythrocytic TRAP from the T9/96 *Pf* strain [35]. Except choosing the antigen, to be a candidate vaccine against malaria, inducing strong T-cell and B-cell responses is also important.

Vaccine using recombinant virus like influenza viruses, poxvirus, syndicate virus, yellow fever virus, adenovirus, and human cytomegalovirus has showed good effects in animal models [36]. Adenovirus-based vaccines were originally developed to induce strong T-cell and B-cell responses, which has an effort like intrinsic adjuvant to eliminate the need for chemical adjuvants in DNA vaccines. However, there is still a big difference between animal tests and human tests. The current method is to combine the vector with the chemical adjuvant. Studies have shown that the titre of antibody induced by adjuvant vaccine is significantly higher than that of the control group without immune adjuvant. Therefore, the rational use of chemical adjuvants will be conducive to the induction of protective T-cell immune response.

Studies showed that prime-boost regimens using the simian adenovirus 63- (ChAd63-) modified vaccinia virus Ankara (MVA) vector expressing the clinically relevant *Pf* ME-TRAP antigen have shown outstanding protective efficacy in mice and macaques [34]. Heterologous prime boosting with ChAd63-MVA has been shown to be a potent inducer of T cells in humans and has demonstrable efficacy when expressed with the pre-erythrocytic ME-TRAP antigen insert [37]. This antigenic insert remains the most promising for a vectored liver-stage vaccine [38]. It is reported that after giving ChAd63 MVA ME-TRAP vaccine, anti-TRAP IFN-*γ* responses in infants were as high as that in adults. IgG responses, mainly composed of IgG1 and IgG3 isotypes, were higher than those in other time after boosting in the vaccines.

Moreover, Gomez et al. [39] inserted the SSP-2 gene into pBSK to obtain pBSK-SSP-2 and recombined it with pMOhyl1 to obtain pMO-SSP-2. After transformation by electroporation into the SL3261 strain, positive strains were screened and cultured. In mice immunized intranasally, serovar Typhimurium constructs secreting SSP-2 stimulated interferon-gamma splenocyte responses [39].

### 2.3. LSA Vaccines

LSA-1 is a 230 kDa protein with a central region containing 86 repeats of the 17-amino-acid sequence EQQSDLEQERLAKEKLQ or minor variations thereof [40]. As the LSA-1 immune response is close to that of naturally transmitted parasites, this antigen has become an attractive vaccine candidate [15, 16], and several clinical trials have tested the effects of LSA-1 vaccines. LSA-NRC, an antigen containing T-cell epitopes from the N and C terminal regions of LSA-1 and several central amino acid repeats, was expressed in *Escherichia coli* and combined with AS01B or AS02A to induce LSA-1-specific cellular immune responses [16]. In addition to LSA-1, the highly conserved LSA-3 protein is a candidate for a subunit vaccine [17]. A vaccine containing the LSA-3 protein elicited a pre-erythrocyte antigen response in the majority of individuals from different age groups [18]. However, its safety, immunogenicity, and efficacy have not been reported on to date [16].

### 2.4. PfSPZ Vaccines

*Plasmodium* SPZs are transmitted to and multiply within the human liver after being carried in the bloodstream when an individual is bitten by a female *Anopheles* mosquito [36]. Therefore, pre-erythrocytic vaccines against malaria need to be highly effective at killing liver SPZs because as little as one SPZ can initiate a malaria infection. For a PfSPZ vaccine to work effectively, it needs to kill SPZs before they arrive at the liver or during their development in this organ. PfSPZ vaccines can be divided into several groups. Attenuated PfSPZ vaccine is the whole parasite attenuated by irradiation. It is reported that sporozoite vaccine can induce T cell against the malaria infection. Moreover, since the expression of IFN-*γ* was related to CD8^+^ T cells, animal studies demonstrated that the vaccine induced high level of PfSPZ-specific CD8^+^ T cells and IFN-*γ*. Compared with heat-inactivated PfSPZ, the frequency of IFN-*γ*-producing CD8+ T cells was much higher in attenuated parasite vaccine. Till now, one of the highest levels (more than 90%) of protection against malaria in humans has been achieved only by immunization with radiation-attenuated PfSPZ. The PfSPZ-chemoprophylaxis vaccine (PfSPZ-CVac) is attenuated by concomitant administration of chemoprophylaxis [3]. It is reported that PfSPZ-CVac achieves similar protective efficacy but with fewer parasites. It is interesting that only 22% (2 of 9) protected volunteers had been induced IFN-*γ*-producing CD8^+^ T cells at the time of CHMI, which is much lower than radiation-attenuated PfSPZ. The difference should be carefully concerned by future studies. Except attenuated by irradiation and using antimalarial drug, gene knockout is another way to make the PfSPZ vaccine. One of the lead candidates is PfSPZ-GA1. This vaccine is a kind of parasite in which the two proteins Pfb9 and Pfslarp were knocked out, which allows liver infection of the parasite without subsequent development in the liver.

Despite the difficulty of this task, one study was successful at achieving 100% protection (in 6 out of 6 subjects) with a radiation-attenuated PfSPZ vaccine [41]. Elsewhere, three doses of a chemoattenuated PfSP vaccine prevented infection in 9 out of 9 (100%) volunteers [3]. These results show that attenuated PfSPZ vaccines show encouraging evidence of efficacy in clinical trials. Till now, the trials of PfSPZ vaccine in Mali confirm efficacy against naturally transmitted parasites, but the dosage, regimen, and route of administration of vaccine are also need to be analyzed. It is shown that PfSPZ vaccine can protect against a heterologous CHMI (3 weeks and 24 weeks) by a 3-dose regimen. Moreover, PfSPZ vaccine was given to 41 adults in Mali. It is reported that 66% of participants were infected, compared with 93% of the control group after five-dose vaccines [42]. With low rate of systemic adverse event, the vaccine was shown to be well tolerated and safe. However, the level of CD4, CD8, and *γδ* T cells showed no difference between the groups, which was different from other studies. The difference in infection rate and protection effect may be due to their regional differences. The infection rate in epidemic areas should be significantly higher than that in nonepidemic areas. Till now, it is easier to make a vaccine that can safely protect short-term travellers. Keeping antibody levels and specific T-cell responses is still a challenge for the development of PfSPZ vaccine.

## 3. Asexual Blood-Stage Vaccines

Clinical signs and symptoms, including recurrent fever, can emerge after *Plasmodium* parasites circulate as blood-stage parasites in the red blood cells of the human body (Figure 2(b)). The presence of these stages (merozoites, rings, trophozoites, schizonts, and gametocytes) causes severe pathology and even death. After entering the human red blood cells from the liver, the surface protein of the *Pf* merozoite can remain in the red blood cell membranes and be exposed directly to the host's immune system. This can stimulate T cells and B cells to produce immune responses. It is reported that *γδ* T cells may produce antibody and perform protective immunity to blood stage of malaria infection. The study has shown that when infected with parasite, IFN-*γ* and IgG2a in *γδ* T-cell-depleted mice were significantly lower than in control mice [43]. Therefore, research on blood-stage malaria vaccines has for a long time been a “hot spot” of vaccine research.

### 3.1. Merozoite Surface Protein 1 (MSP1) Vaccines

MSP1 is located on the merozoite surface where it plays a key role in erythrocyte invasion. PfMSP1, which is a blood-stage antigen, is hydrolyzed into PfMSP1_83_, PfMSP1_28_, PfMSP1_38_, and PfMSP1_42_ before merozoite invasion of the erythrocytes. During invasion, PfMSP1_42_ is processed into MSP1_33_ and MSP1_19_ [19]. PfMSP1_19_ contains the structural domain of the epidermal growth factor and is rich in cysteine residues. It can become anchored to the merozoite surface and combine with the erythrocyte surface receptor band 3 protein. In trials conducted to test PfMSP1-based vaccines, all the candidate vaccines were found to be safe and highly immunogenic [44]. Some reports have indicated that MSP1_19_ has no efficacy in terms of reducing the parasite multiplication rate in homologous controlled human malaria infections or natural infections [20, 45, 46]. Alaro et al. [21] produced recombinant *Pf*MSP1_19_ fused to the N-terminus of the *Pf* merozoite surface protein 8 with the low-complexity Asn/Asp-rich domain (rPfMSP8) lacking. Immunization of mice with the chimeric rPfMSP1/8 vaccine elicited strong T-cell responses to the conserved epitopes associated with the rPfMSP8 fusion partner. PfMSP1/8-specific rabbit IgG was shown to potently inhibit the *in vitro* growth of FVO and 3D7 strains of blood-stage *Pf* parasites [22]. Since people can be infected with different kinds of parasites in different regions, a wide range of epidemiological surveys is also needed. By analyzing the distribution of certain strains in malarial regions and the changes over time, it can help people develop specific vaccines for specific areas (although the vaccine may not be effective elsewhere). It is reported that MSP1_19_ in FVO or FuP strain vaccines may be more effective than 3D7 strain in Malian because the most common parasites strain causing malaria are from FVO or FuP [47]. Moreover, some kinds of bioinformatics software like BioStructMap can be used in the research screening. By studying the selection in different regions and the diversity of structural patterns, the bioinformatics software can be used to analyze the good MSP1_19_ vaccine candidates of the parasite.

### 3.2. Apical Membrane Antigen 1 (AMA-1) Vaccines

AMA-1 is expressed in the sporozoite, hepatic, and erythrocytic stages [23] where it plays an essential role in parasite survival [45]. The AMA-1/AS02 blood-stage vaccine demonstrated no overall efficacy, but sieve analysis revealed allele-specific efficacy against the vaccine strain, suggesting that AMA-1 could be a useful inclusion in a multicomponent vaccine [46]. In a series of trials, experimental malaria vaccines formulated with AMA-1 and an AS01B or AS02A adjuvant system had demonstrable safety, tolerability, and immunogenicity [20–22]. The AMA-1 molecule has 16 conservative cysteine residues, which divides the protein into three substructures. Among them, AMA-1 (III), which is located in the C-terminal region, is genetically conserved and has been considered as a candidate vaccine component. Takala et al. [47] found that *Pf* chimeric protein 2.9 (PfCP-2.9), which consists of MSP1_19_ and AMA-1 (III) sequences, induced parasite-inhibitory antibodies in rabbits and monkeys. The safety and immunogenicity of PfCP-2.9 formulated with the novel Montanide ISA 720 adjuvant were tested, and all the candidate vaccines were shown to be safe and immunogenic [47].

The development of malaria vaccine is a long and arduous process because people can be infected with different kinds of malaria strains. Considering the complexity of malaria antigens, if the parasite proteins obtained from people are different from the ones used for vaccination, it may lead to the immune response that may be less effective or even ineffective. Therefore, an ideal candidate vaccine should consider the effect of alleles on immunogenicity. A method to solve the problem is that AMA-1-C1 (a mixture of AMA-1-FVO and AMA-1-3D7 recombinant proteins) is designed to protect against a majority of parasite genotypes. But the effective titres of antibodies to the proteins and the integrity of the protein by long-term storage are also need to be concerned. Now, some research studies try to generate a vaccine that targets the conserved regions of the AMA-1, but such a vaccine is not used in clinical till now.

### 3.3. Rh5 Vaccines

The blood stages of a malaria infection cause morbidity and mortality in humans. Hence, vaccines targeting these stages aim at protecting the human host against clinical disease and death. Till now, extensive efforts usually focused on a small number of well-studied merozoite antigens targeting naturally acquired immunity to induce antibodies that block erythrocyte invasion [36]. Rh5, as the most important candidate for the blood-stage vaccine, rapidly invades red blood cells by forming complex with cyrpa and ripr and binding with basigin of red blood cells. Therefore, Rh5-targeted vaccine can prevent pathogens from invading the human body. At present, the 3D image information of the protein complexes has been studied. Structural analysis of the complexes will contribute to screen the target position of complexes and development of the vaccines. In addition, studies have found that p113 can interact with Rh5nt directly, so it also can be developed as a candidate vaccine [23]. Moreover, a group of human monoclonal antibodies (mabs) against pfrh5 were isolated from the peripheral blood B cells of the volunteer. One subgroup of mabs neutralizes the antibody while the other subgroup of monoclonal antibodies plays an important role by slowing down the binding speed between Rh5 and the receptors of red blood cells, thus making the antibodies to Rh5 more effective [24]. To date, only a few candidate vaccines (such as the RH5.1/AS01B vaccine) have been allowed to be used in phase I/IIa clinical trials. Clearly, more data on the safety, immunogenicity, and efficacy of blood-stage vaccines are still required [25].

## 4. Vaccines Targeting the Sexual and Mosquito Stages

Transmission-blocking vaccines (TBVs) are designed to control the transmission of malaria parasites from human hosts to the mosquito vectors (Figure 2(c)). In the 1950s, Hill et al. [48] first demonstrated the induction of transmission-blocking immunity in chickens by repeated immunization with Plasmodium gallinaceum–infected red blood cell. Since then, significant advances have been made in TBV development. Subsequent studies have shown that antigens expressed later in parasite development after fertilization can be targeted [49]. Sherrard et al. found that TBVs could reduce parasite density in the mosquito salivary glands, thereby enhancing pre-erythrocytic vaccine efficacy [50]. The leading TBV candidates include the Pfs25, Pfs48/45, and Pfs230 which have shown transmission-blocking immunity in model systems in different stages of development. [51]. During the stage of transmission, the T cells usually impact on it. It is reported that the lysate of mature gametocyte-infected RBCs leads to the T-cell proliferation and the expression of IFN-*γ*. Moreover, T cells associated with B cells may enhance the immune response during the malaria infection. B-cell knockout mice induced a CD4^+^T Th1-like response compared with Th2 cells in the control group, which means that B cells play a role in the regulation of CD4^+^T subset responses [36].

### 4.1. Pfs25

Pfs25, a 25 kDa sexual-stage antigen present on the surfaces of *Pf* mosquito-stage gametes and zygotes, has four epidermal growth-like factor structural domains, a large number of cysteine residues, and a complex polymer structure [26, 52]. In 2008, the National Institutes of Health, USA, completed a phase I clinical trial using Pfs25 as the antigen [27]. Pfs25 fused to IMX313 and expressed in ChAd63 and MVA viral vectors led to a qualitatively improved antibody response compared with Pfs25 alone, as well as to significantly higher germinal center responses [28]. Lee et al. [26] reported that Pfs25 expressed in baculovirus and *Pichia* showed promise for TBV development. Interestingly, Scally et al. [51] reported that six crystal structures of Pfs25 in complex with the antibodies that were elicited by immunization had good specificity associated with two distinct immunogenic sites on the Pfs25 protein.

### 4.2. Pfs48/45

Pfs48/45 is expressed on the surfaces of gametocytes (from stage III onwards) and gametes and is bound to the parasite membrane through a GPI anchor. It forms a stable complex with Pfs230, another important TBV candidate. Animal studies have shown that transmission-blocking antibodies can be stimulated by immunization with Pfs48/45 [29]. Human antibodies against Pfs48/45 and Pfs230 proteins have been investigated in a study in central Ghana [53], where their seroprevalence was found to be 74.7 and 72.8%, respectively [29]. Singhet al. [54] reported that SpyCatcher-R0.6C and SpyCatcher-6C in Pfs48/45-based virus-like particle vaccines were responsible for significantly increased antigen immunogenicity when Montanide ISA 720 VG was used as the extrinsic adjuvant.

Current data suggest that in addition to Pfs25 and Pfs48/45, identifying new target antigens and/or combinations thereof could be a worthwhile strategy for future vaccine research. Structural biology can be used to predict the defensive epitopes, genomics, and proteomics for novel vaccines. Moreover, safe adjuvants that can effectively boost the immune system should be also fully concerned.

## 5. Conclusions

We have described three types of malaria vaccine in this review (Table 1). The completed phase III clinical trial of RTS,S, the most promising vaccine to date, showed that the vaccine's efficacy was limited and that its effect was geographically regional. There is no doubt that more research is needed for malaria vaccine development. Using new tools to identify new target antigens is one of the most important ways in future. Moreover, to increase the immune system response, more kinds of adjuvant systems should be developed to speed up the development of a new generation of malaria vaccine.

In this limited review, we are not covering all approaches to malaria vaccine development, or most of the critically important work on the development of vaccines against *P. vivax*, the second most important cause of malaria. The most difficult part for the development of *P. vivax* vaccines is that the parasite is not tractable to *in vitro* culture. The lack of a method for the continuous culture of *P. vivax* blood stages makes it difficult to identify synergistic antigens in blood stage. Till now, some groups have successfully established short-term *P. vivax* culture, and more tools for parasite culture are still needed for further development of vaccines against *P. vivax*.

## Figures and Tables

**Table 1:** List of *Plasmodium* vaccines in pre-erythrocytic stages, blood stages, and transmission-blocking stages.

Vaccine group	Vaccine name	Vaccine type	Malarial antigen targeted	Mechanism of action	References
Pre-erythrocytic vaccines	PfCSP vaccines	Subunit vaccines	PfCSP	Antibodies to PfCSP block the sporozoite invasion of liver cells	[5, 6]
	RTS,S/AS01 and RTS,S/AS02		Hepatitis B surface antigen and the central repeat and C-terminal regions of CSP	Protective immune responses after vaccination with RTS,S are dependent primarily on antibody responses against the central repeat region	[7–11]
	PfCSP bacteria vaccines		PfCSP	Bacteria need assistance to stimulate the innate immune system	[12, 13]
	TRAP vaccines	Subunit vaccines	SSP-2	Antibodies to SSP-2 block the invasion of mosquito salivary glands and hepatocytes	[14]
	LSA vaccines	Subunit vaccines	LSA-1/LSA-3	Elicit a pre-erythrocyte antigen response in the majority of individuals from different age groups	[15–18]
	PfSPZ vaccines	Live attenuated vaccines	PfSPZ	Antibodies to PfSPZ block the parasite arrival to the liver or during their development in this organ	[3]

Asexual blood-stage vaccines	MSP1 vaccines	Subunit vaccines	MSP1_42_/MSP1_38_/MSP1_83_	Antibodies to MSP1 to block the parasite invasion of erythrocyte	[19]
	AMA-1 vaccines	Subunit vaccines	AMA-1	AMA-1 plays an essential role in parasite survival. Antibodies to AMA-1 may kill the parasite	[20–22]
	Rh5 vaccines	Subunit vaccines	Rh5	Antibodies to Rh5 block the parasite invasion of erythrocyte by forming complex with cyrpa and ripr	[23–25]

Transmission-blocking vaccines	Pfs25 vaccines	Subunit vaccines	Pfs25	Pfs25 is the target on which the parasite survives and interacts with the mosquito midgut. Antibodies to Pfs25 control the transmission of malaria parasites from human hosts to the mosquito vectors	[26–28]
	Pfs48/45 vaccines	Subunit vaccines	Pfs48/45 C-terminus	Pfs48/45 is the target on which male gamete attaches to female gamete. Antibodies to Pfs48/45 can induce transmission-blocking antibody response during infection	[29]
